# University Day

**Published:** 1901-03

**Authors:** 


### UNIVERSITY DAY.

THE University of Buffalo has adopted February 22d as University Day and celebrated it with appropriate proceedings this year. In this it follows the usual course of colleges and universities in establishing a custom which becomes an important event on the calendar. In founding the day the council and faculty have chosen appropriately, since the first public college work was done February 24, 1847. Washington's birthday being a legal holiday and falling so close to the anniversary of the beginning of the first course of medical lectures makes the action of the boards a befitting one.

The exercises began in the morning at the Star Theatre and, in true college fashion, the collegians heralded the joyous occasion. The auditorium of the theater was handsomely decorated with the national and college colors, banners and insignia. The class fraternities were present in full force and fine array, and the theater was well filled when the ceremonies began.

Promptly at 11 o'clock the council and faculties of all the departments marched upon the stage amidst rounds of applause. The exercises began with a prayer by the Rev. O. P. Gifford, after which the chancellor of the University, the Hon. James O. Putnam, made a short address, taking for his theme the history of the University. The University double quartette then sang a Pickanniny lullaby, which was warmly applauded.

The chancellor introduced Dr. Rush Rhees, president of the University of Rochester, the subject of whose address was "Success." The University mandolin club then gave a selection from the Fortune Teller, after which America was sung and the benediction pronounced. A fine selection by the orchestra ended the exercises.

Dr. Matthew D. Mann, dean of the medical faculty, and Mrs. Mann, tendered a reception to the faculty and students from 4 to 6 in the afternoon.

The evening was appropriated by the alumni of the medical department in giving their third mid-winter reunion, which took place in the Alumni Hall of the University building. At 8 o'clock the Rev. Frederick J. Hillig, S. J., professor of sciences at Canisius College, demonstrated the physical and physiological effects of ultra-violet light. In the course of his demonstration he introduced many interesting experiments, among which was the exhibition of Professor Zickler's new light-electric telegraphy (wireless) and Professor Strebel's apparatus for killing bacteria with ultra-violet light. These experiments were successfully conducted, were entertaining as well as instructive and the professor was frequently interrupted with demonstrations of applause.

Dr. M. Hartwig, president of the Buffalo Academy of Medicine, followed with a short address on the German Doctor, interspersing his remarks with witty stories of professional life in the fatherland. He spoke of the "Staats Examen," giving a brief description of it and strongly endorsing its analogue, which is being established by many states in this country.

Professor Roswell Park was next introduced and gave some interesting reminiscences incident to professional study in Europe. In the course of his remarks he referred to the school of Salernum, which became so celebrated during the middle ages, but of which there is now not a vestige remaining by which even its location can be determined with certainty. He said the effect of this school upon future medical history was very important.

Dr. Frederick C. Busch, professor of physiology, delivered an interesting address upon Student life in Switzerland, describing especially his recent visit to the University of Berne.

Dr. A. W. Bayliss, vice-president of the alumni association, presided in the absence of the president, Dr. D. W. Harrington, who was ill. Dr. Bayliss read a telegram of regret from Dr. W. H. Bergtold, of Denver, Col. A vote of thanks was tendered to the speakers and committees and the large audience then adjourned to the library where refreshments were served.

The exercises were interspersed with orchestral music and the entire proceedings were eminently befitting the occasion and bespoke in a most emphatic manner the success of the great University. Such a day as thus imperfectly outlined cannot but serve to stimulate closer friendship between the University and the community.

The committees who arranged the several exercises were composed as follows: On the part of the University faculties—Dr. Eli H. Long, of the medical department; Dr. Willis G. Gregory, of the department of pharmacy; E. Corning Townsend, of the department of law and Dr. W. C. Barrett, of the department of dentistry. On the part of the Alumni Association, Dr. Albert T. Lytle, Dr. Grover W. Wende, Dr. H. U. Williams, Dr. F. W. Barrows and Dr. T. H. McKee. These committees deserve great credit for the splendid manner in which their work was done.

